# Behavioral evidence of the functional interaction between the main and accessory olfactory system suggests a large olfactory system with a high plastic capability

**DOI:** 10.3389/fnana.2023.1211644

**Published:** 2023-10-16

**Authors:** Zacnite Mier Quesada, Wendy Portillo, Raúl G. Paredes

**Affiliations:** ^1^Instituto de Neurobiología, Universidad Nacional Autónoma de México (UNAM), Querétaro, Mexico; ^2^Escuela Nacional de Estudios Superiores, Unidad Juriquilla, Universidad Nacional Autónoma de México (UNAM), Querétaro, Mexico

**Keywords:** olfactory system, accessory olfactory system, main olfactory system, neurogenesis, paced mating

## Abstract

Olfaction is fundamental in many species of mammals. In rodents, the integrity of this system is required for the expression of parental and sexual behavior, mate recognition, identification of predators, and finding food. Different anatomical and physiological evidence initially indicated the existence of two anatomically distinct chemosensory systems: The main olfactory system (MOS) and the accessory olfactory system (AOS). It was originally conceived that the MOS detected volatile odorants related to food, giving the animal information about the environment. The AOS, on the other hand, detected non-volatile sexually relevant olfactory cues that influence reproductive behaviors and neuroendocrine functions such as intermale aggression, sexual preference, maternal aggression, pregnancy block (Bruce effect), puberty acceleration (Vandenbergh effect), induction of estrous (Whitten effect) and sexual behavior. Over the last decade, several lines of evidence have demonstrated that although these systems could be anatomically separated, there are neuronal areas in which they are interconnected. Moreover, it is now clear that both the MOS and the AOS process both volatile and no-volatile odorants, indicating that they are also functionally interconnected. In the first part of the review, we will describe the behavioral evidence. In the second part, we will summarize data from our laboratory and other research groups demonstrating that sexual behavior in male and female rodents induces the formation of new neurons that reach the main and accessory olfactory bulbs from the subventricular zone. Three factors are essential for the neurons to reach the AOS and the MOS: The stimulation frequency, the stimulus’s temporal presentation, and the release of opioids induced by sexual behavior. We propose that the AOS and the MOS are part of a large olfactory system with a high plastic capability, which favors the adaptation of species to different environmental signals.

## Introduction

Olfaction is an essential sensory system in many species of mammals that contributes to the survival of the individual and the species. For example, in rodents, this system is crucial for foraging, identifying predators, and recognizing conspecifics that influence social and reproductive interactions, including parental and sexual behavior. Early anatomical evidence suggested the existence of two anatomically distinct chemosensory systems: The accessory olfactory system (AOS) and the main olfactory system (MOS) (Scalia and Winans, 1975; Halpern and Martinez-Marcos, 2003).

The olfactory receptor cells of the AOS are located within the vomeronasal organ (VNO). These receptors receive and identify the odor signals, usually known as pheromones, which are chemicals released by an animal that elicit a response, physiological and/or behavioral, in a conspecific. Axons arising from the second-order mitral/tufted transmit information to the bed nucleus of the stria terminalis, to the bed nucleus of the accessory olfactory tract, and the medial and the posteromedial part of the cortical nuclei of the amygdala (Mucignat-Caretta et al., 2012). The amygdala sends projections to the accessory olfactory bulb (AOB) through the stria terminals (Raisman, 1972). The bed nucleus of the stria terminalis connects with the mitral cell layer of the AOB, and the amygdala nuclei send their fibers to the granule cells in the AOB; review in Mucignat-Caretta et al. (2012).

The polysynaptic AOS is sexually dimorphic in the rat, with males having more neurons in several segments, including the VNO, the AOB, the BNST, and the MPOA/AH (Bleier et al., 1982; Segovia and Guillamon, 1993; Guillamon and Segovia, 1997). Neurons from the BNST send direct and indirect fibers to the forebrain and brain stem endocrine and motor visceral nuclei, resulting in the correspondingly hormonal and motor responses.

The MOS has its receptors in the main olfactory epithelium (MOE; Xu et al., 2000). Olfactory receptor neurons in the MOE send their axons to glomeruli located on the main olfactory bulb (MOB) glomerular layer, where they synapse with dendrites of mitral/tufted cells. MOB mitral and tufted cells project to the olfactory cortex, which is integrated by the anterior olfactory nucleus, the anterior and posterior piriform cortex, the olfactory tubercle, the lateral entorhinal cortex, the medial amygdaloid nucleus, and the anterior and posterolateral cortical amygdaloid nucleus (Raisman, 1972; Kevetter and Winans, 1981; Pro-Sistiaga et al., 2007; Kang et al., 2009; Sosulski et al., 2011; Thompson et al., 2012; Imamura et al., 2020). The MOB receives inputs from the locus coeruleus and has connections from the dorsal and median raphe nuclei (McLean and Shipley, 1987; Matsutani and Yamamoto, 2008). Projections from the MOB and AOB converge in the amygdala, AOB mitral cell axons synapse in the deep region. The MOB mitral axons are located in the superficial layer (Kang et al., 2009). Thus, the amygdala processes information from both systems (Licht and Meredith, 1987). The amygdala sends projections to the preoptic, ventromedial, and ventromedial premammillary nuclei (Mucignat-Caretta et al., 2012).

Based on the different pathways and connections for both systems and some early behavioral and physiological studies, it was assumed that they carry different types of information. It was postulated that the AOS was crucial for the detection of pheromones, especially the non-volatile sexually relevant olfactory cues in rodents influencing reproductive behaviors and neuroendocrine functions (Halpern, 1987; Bargmann, 1997; Tirindelli et al., 1998; Keverne, 1999) such as intermale aggression, sexual preference, maternal aggression, pregnancy block (the Bruce effect), puberty acceleration (Vandenbergh effect), induction of estrous (Whitten Effect) and sexual behavior. On the other hand, the MOS was proposed to detect volatile odorants related to food, giving the animal information about the environment (Laurent, 1999; Leon and Johnson, 2003).

### Early evidence of the participation of the MOS in pheromone processing

The anatomical and functional subdivision postulated initially was not evident, and there were clear examples that it was not as straightforward as initially conceived. Early studies demonstrated that ablation of the olfactory bulbs (OB) affected different social behaviors in several rodents, including chemoinvestigation, olfactory preference, and sexual behavior (Petrulis, 2013). These alterations could be affected by previous exposure to odors from conspecifics and prior sexual experience. Studies in other species also suggested that pheromonal stimulation was not dependent on the AOS. For example, in the domestic pig, the pheromone androstenone facilitates the attraction to the male as well as the receptive posture of the female. Surgical cement was applied to the VNO to evaluate if this structure mediates the effects of androstenone in female pigs. No effects were observed in the females with the vomeronasal organ blocked. That is, they were not different from control subjects, indicating that the vomeronasal organ, hence the AOS, is not involved in processing the effects of androstenone in sexual response in female pigs (Dorries et al., 1997). Similar results were described in rabbits (Table 1). In this species, the release of a pheromone by the belly of the mother induces nipple search and attachment. A study in newborn rabbits demonstrated that the surgical removal of the vomeronasal organ did not affect the ability of the pups to obtain milk. On the other hand, irrigation of the nasal mucosa with zinc sulfate (ZnSO4) eliminated the response to the pheromone and a conditioned odor for citral, suggesting that pheromonal processing in response to the release of suckling in rabbits is not mediated by the vomeronasal organ (Hudson and Distel, 1986). Later, Schaal and colleagues identified a volatile compound, 2 MB2, from the rabbit milk, which induces nipple search. This pheromone is specific for the species, and they name it mammary pheromone because it is produced the novo in the mammary tract (Schaal et al., 2003).

**Table 1:** Different examples of the early evidence implicating the involvement of the main olfactory bulb (MOB) in the processing of chemosensory relevant cues and of the functional interaction between the accessory olfactory bulb (AOB) and the MOB in the processing of different stimuli associated with reproduction.

Species	Procedure	Effect	References
Domestic Pig	Surgical cement applied to the VNO	No alteration in attraction to odors or receptive posture	Dorries et al. (1997)
Rabbits	Surgical removal of the VNO in pups	Did not affect the ability to obtain milk mediated by pheromones	Hudson and Distel (1986)
Hamsters	Surgical removal of the VNO	Did not affect scent marking behavior	Johnston (1992)
	Vomeronasal organ removal	Did not affect Fos Expression induced by Pheromones	Swann et al. (2001)
Ferret	Subjects exposed to odors from estrous females after Testosterone propionate administration	Increased Fos expression in the MOB but not the AOB	Kelliher et al. (1998)
	Major histocompatibility complex Class I peptide ligands	Detected by the MOB and AOB	Kelliher (2007)
Mice	Late pregnant female mice exposed to pups	Similar levels of Fos expression in the MOB and AOB	Martinez-Garcia et al. (2009)
	High-resolution functional magnetic resonance imaging to evaluate activation to odors	Common odorants induced strong activation of MOB but weak in the AOB. Urine odor induces high activation of the MOB and AOB	Xu et al. (2005)
	Mice housed with same sex or opposite sex from weaning to 6 months	Expression of chemoreceptors in MOE and VNO more divergent if housed with members of the same sex	van der Linden et al. (2018)
Rat	Direct projection from the anterior AOB to the dorsal MOB	Both systems are highly interconnected	Vargas-Barroso et al. (2016)

Studies on hamsters showed similar results. Removal of the vomeronasal organ in female hamsters reduced the frequency of ultrasonic calling in females when exposed to odors from conspecifics but did not affect scent marking behavior (Johnston, 1992). Administration of ZnSO4 into the nasal mucosa reduces ultrasonic calling, flank, and vaginal marking when exposed to odors from a conspecific. The same treatment impaired the ability to localize buried food. These results suggested that the MOS mediated scent-marking behavior in response to odors from conspecifics (Johnston, 1992). A similar experiment in male hamsters demonstrated that the MOS mediates scent marking and does not require the participation of the vomeronasal organ (Johnston and Mueller, 1990). A series of studies in ungulates demonstrated that the MOS is involved in maternal behavior, offspring recognition at birth, and the so-called male effect, which is the reactivation of the gonadotropic axis when a male is introduced in a group of females in the anestrous season (Keller and Levy, 2012) and references therein. Together, these studies demonstrated that the chemosensory relevant cues, pheromones, did not necessarily produce their actions through the activation of the AOS but an interaction of both systems could explain the results (Table 1).

### Immediate early gene studies

The immunocytochemical visualization of different immediate-early genes, through their nuclear protein products, such as c-fos and c-jun, have been used to study how several stimuli, including olfactory stimulation, activate brain circuits. Early studies described that mating significantly increased Fos expression in different brain regions of the AOS compared to control animals. These regions included the medial AMG, the BNST, the MPOA/AH, and the ventromedial hypothalamus (VMH) (Baum and Everitt, 1992; Baum et al., 1994; Newman et al., 1997). Moreover, exposure to sexually relevant odors also induces higher Fos expression in regions of the AOS in gerbils (Heeb and Yahr, 1996), hamsters (Fernandez-Fewell and Meredith, 1994), and rats (Bressler and Baum, 1996; Coolen et al., 1997).

In the early studies using Fos expression to determine the involvement of brain regions in the processing of chemosensory relevant cues, little attention was given to the MOS. For example, the analysis concentrated on the AOS in the first study in which Fos expression was used to evaluate if brain areas were activated in response to exposure to male odors. Although no data was shown, the authors mention that “No fos-like immunoreactivity was detected in the MOB, hippocampus, caudate putamen, or globus pallidus” (Dudley et al., 1992). In another study in Golden Hamsters, males were allowed to mate with a receptive female, exposed to vaginal fluids, or placed alone in a clean cage. The vomeronasal organ was removed (VNX) in half of the animals and left intact in the other half. The few VNX subjects that mated showed similar patterns of Fos expression to that of intact animals but with less intensity in the AOS. Interestingly, the authors described that in the MOS, stimulated and unstimulated animals showed low levels of Fos expression. However, it is unclear if they made statistical comparisons (Fernandez-Fewell and Meredith, 1994), again confirming the little attention given to the MOB in early Fos studies.

We also did several studies using Fos expression to evaluate the participation of different brain regions in the processing of chemosensory-relevant olfactory cues. For example, we tested if the increase of Fos expression in response to odor from estrous females is hormone dependent. Male and female rats were gonadectomized and supplemented with testosterone propionate (TP) or oil and exposed to odors from estrus females. The increase in Fos expression was seen only in subjects treated with TP (Paredes et al., 1998). In another study, we evaluated Fos expression in males without sexual experience, copulating males, and non-copulating males when exposed to estrous bedding. Non-copulating rats are males who do not mate even if tested repeatedly with receptive females. We found an increase in Fos expression in the mitral cell layer of the AOB in the three groups after exposure to estrous bedding. Only the group of copulating males showed an increase in Fos expression in the structures of the AOS. Males with no sexual experience only showed increased Fos response in the MPOA. In the non-copulating males, we did not observe an increase in Fos expression in any region of the AOS. We proposed that non-copulating males have reduced sexual motivation, reflected by a lack of Fos response in the AOS when exposed to estrous odors (Portillo and Paredes, 2004). In both studies, we looked at one area of the granular cell layer of the MOB, chosen arbitrarily, but no systematic analysis of the MOB was done. The straightforward explanation back then, when our results were compared to the clear effects seen in other species, see below, was species differences. We also evaluated Fos expression in sexually sluggish male rats; those that take longer to display mounts, intromissions, and ejaculations and show a reduced number of these behavioral parameters. We found that the activation of the AOS was similar to that of copulating males (Portillo et al., 2006). In that study, we did not evaluate Fos expression in the MOB. In another study, we assessed the contribution of the MPOA-AH to olfactory preference and the activation of the AOS in response to odors from estrous females because it is well established that lesions of the MPOA-AH permanently eliminate the expression of male sexual behavior in several species [review in Paredes (2003)]. We found that before the lesion, males showed a strong preference for estrus bedding, but after the lesion, they showed no preference between estrus and anestrus bedding. Fos expression was similar in intact and lesioned males in areas of the AOS, suggesting that changes in olfactory preference are not associated with alterations in the processing of olfactory cues by the AOS (Hurtazo and Paredes, 2005). As in the previous study, we did not evaluate Fos expression in the MOB, but our interpretation nowadays would be different, suggesting that no alterations in Fos expression were found because the MOS also processes the olfactory information.

So, early studies of Fos detection found no differences in the MOS, or since the expression was not as evident as that observed in the AOS, it was assumed that it did not participate in pheromone processing. However, we should remember that the absence of cellular Fos expression in a particular brain region in response to a specific stimulus does not necessarily mean that neurons/glia of that brain area are not involved in processing that stimulus.

### Interaction between the accessory and main olfactory systems

Some of the early studies using Fos expression, demonstrating the participation of the MOS in the processing of pheromones, were done in hamsters and ferrets. Swann et al. (2001) removed the vomeronasal organ or administered ZnSO4 to the MOE of male hamsters exposed to vaginal secretions of ovariectomized females hormonally treated with estradiol and progesterone. The administration of ZnSO4 eliminated the increase in Fos expression in the AMG, the BNST, and the MPOA-AH induced by exposure to female pheromones. On the other hand, removing the vomeronasal organ did not affect the increase in Fos expression induced by pheromone exposure, suggesting that the MOS plays a crucial role in processing pheromones (Swann et al., 2001). A ferret study showed that testosterone propionate increased the expression of Fos in the MOB but not the AOB when subjects were exposed to odors from estrous females (Kelliher et al., 1998). The interaction between the MOS and AOS has also been observed in pregnant females. The MOB and the AOB showed similar levels of Fos expression in late pregnant female mice when exposed to pups (Navarro-Moreno et al., 2020). In fact, it was proposed that the AOS is involved in recognizing conspecific gender while the MOS allows an individual, male or female, to identify the scents of conspecifics (Martinez-Garcia et al., 2009). On the other hand, it has been proposed that no general rules can be applied to olfactory detection, assuming that the AOS detects pheromones while the MOS detects general odorants; in each case, both systems can have a specific response (Restrepo et al., 2004).

After the early studies, it was evident, as described by different research groups (Table 1), that both olfactory systems are integrated and regulate olfactory responses to pheromones (Brennan and Zufall, 2006; Mucignat-Caretta et al., 2012; Baum and Bakker, 2013) and interact in the control of mate recognition and sexual behavior (Keller et al., 2009). For example, both systems can detect major histocompatibility complex Class I peptide ligands providing similar genetic information (Kelliher, 2007).

This interaction between the two olfactory systems starts at the level of the olfactory sensory receptors. For example, the olfactory stimulation perceived by mice can change the turnover of the chemoreceptors in the MOE and the VNO. In an interesting study male and female mice were housed with members of the same or opposite sex from weaning until they were 6 months old. The authors evaluated the expression of chemoreceptors and other genes by quantitative PCR for the presence or absence of tissue and sex-specific markers (Santoro and Jakob, 2018). They found that the expression frequency of chemoreceptors in the MOE and the VNO are more divergent if they are housed with members of the same sex than if males and females are housed together, demonstrating that there are important differences in the olfactory sensory receptors depending on how mice are housed after weaning (van der Linden et al., 2018).

A series of anatomical and functional studies also demonstrated that both systems are highly interconnected. Larriva and coworkers demonstrated the existence of a direct projection from the anterior AOB to the dorsal MOB (Vargas-Barroso et al., 2016). They suggested that the olfactory limbus is a site where odorant and pheromone stimulation could be initially processed (Vargas-Barroso et al., 2016). Moreover, exploring neutral odorants or chemosignals of conspecifics activates the circuits originating in the MOB and AOB (Pardo-Bellver et al., 2022).

In another study, high-resolution functional magnetic resonance imaging (fMRI) was used to evaluate the activation of the MOB and AOB to different odors. Common odorants induced a strong activation of the MOB and a weak response in the AOB. On the other hand, urine odor induced a higher activation of the MOB and the AOB, indicating that both olfactory systems respond to volatile compounds (Xu et al., 2005). From the above described information, it is clear that the original subdivision of the AOS and MOS has anatomical interconnections and overlap in the processing of chemosensory information, having a complementary role.

### Transgenic mice

The relevance of the MOB and AOB on reproductive behaviors has also been evaluated using mutant mice. One of the genes involved in chemosensory transduction in the main olfactory epithelium is the cyclic nucleotide-gated channel subunit 2 (Cnga2). Cells that express Cnga2 are distinguished by expression of type-3 adenylate cyclase (Adcy3) and are classified as Adcy3 positive or type A and negative or type B. Activation of Adcy3 increases intracellular cAMP. High levels of cAMP induced by odorant stimulation activate the cyclic nucleotide-gated channel that induces membrane depolarization and action potentials (Brunet et al., 1996). Adcy3 is not expressed in the vomeronasal organ. Trinh and Storm demonstrated that Adcy3 mutant mice can detect volatile odorants via the vomeronasal organ (Trinh and Storm, 2003). Cnga2 is a component of the olfactory signal transduction pathway in the MOB and is not expressed in vomeronasal neurons (Berghard et al., 1996). Cnga2 null mice are anosmic, show no preference for female urine, and do not mate or fight (Mandiyan et al., 2005). Cnga mutant males do not sniff the anogenital or facial area of sexually receptive females, and they do not attempt to mount them (Mandiyan et al., 2005; Bepari et al., 2012). They also show reduced aggressive behaviors (Mandiyan et al., 2005).

The transient receptor potential cation channel (Trpc2) is important for calcium signaling in the vomeronasal organ and is the primary signal transduction in the vomeronasal epithelium. Trpc2 is expressed in the vomeronasal organ, testis, brain, spleen, heart, and thyroid cells (Miller, 2014). Trpc2 regulates pheromone-induced signaling in rodent VNO. However, Omura and Mombaerts (2014) demonstrated that cells in the main olfactory epithelium also express Trpc2, and they identified two types of Trpc2-positive cells, type A and B. They also found that some Trpc2-positive neurons project axons to the glomeruli of the main olfactory bulb. The authors conclude that MOB-positive cells for Trpc2 had properties of olfactory sensory neurons and vomeronasal sensory neurons (Omura and Mombaerts, 2014).

Deficient Trpc2 knockout mice show reduced electrophysiological responses to pheromones in the vomeronasal organ (Leypold et al., 2002). Male and female mice have alterations in sex discrimination and sexual, aggressive, and parental behaviors. Trpc2 knockout male mice have normal levels of testosterone. Still, they do not display pheromone-induced aggression toward other males. When Trpc2 knockout males have access to a female, they show courtship and mount them, but this behavior was directed to both males and females (Leypold et al., 2002; Stowers et al., 2002). Thus, Trpc2 knockout did not prefer males or females and mate with equal frequency with both sexes (Kimchi et al., 2007).

Trpc2 deficient females have regular estrous cycles and estradiol serum levels; their maternal behavior is similar to wild-type dams but display lower levels of maternal aggression in response to male intruders (Leypold et al., 2002). Trpc2 knockout females display male-like behaviors. Thus, they exhibit higher levels of anogenital sniffing toward the male and mounting and pelvic trusting behavior directed to male and female stimulus mice. Male-like sexual behavior of the Trpc2 knockout females towards other females was similar to the sexual behavior observed by heterozygous and mutant males (Kimchi et al., 2007). Kimchi and coworkers also evaluate courtship behavior through solicitation and ultrasonic vocalizations. Male solicitation involves raising the female rear with their snout, and during the interaction, males emit ultrasonic vocalizations at high frequencies (110 kHz). Trpc2 knockout males and females display anogenital investigation and ultrasonic vocalizations in the presence of same-sex conspecifics (Kimchi et al., 2007). Interestingly, adult male mice did not mate with juveniles because this behavior is inhibited by the pheromone exocrine-gland secreting peptide 22 (ESP22) secreted from juvenile mice’s lacrimal glands. Trpc2-deficient male mice cannot recognize ESP22 pheromone and mate with prepubescent females even when they have access simultaneously to sexually receptive adult females (Ferrero et al., 2013).

G coupling protein G_ao_ and members of the V2R receptor family are necessary for the sensory function of basal vomeronasal sensory neurons, and G_ai2_ and V1R receptors expressed in apical vomeronasal sensory neurons modulate aggression directed to pups, parental care in males and maternal aggression (Shinohara et al., 1992; Ryba and Tirindelli, 1997). G_ao_ conditional null male mice showed lower levels of male-to-male territorial aggression. Lactating females with this mutation did not show aggression toward males and did not have alterations in maternal behavior evaluated by pup retrieval (Chamero et al., 2011). Female mice mutants for G_ao_ had alterations in their timing of puberty onset. In response to male urine exposure, G_ao_ females had fewer days in estrous and proestrus and presented less regular estrous cycles. However, G_ao_ females had normal estradiol and progesterone levels and fertility rates (Oboti et al., 2014). Oboti and collaborators also evaluated if the exposure to an unfamiliar male-induced pregnancy block, mutants for G_ao_ exposed to urine from unfamiliar males, did not increase pregnancy failure. After exposure to a male, G_ao_ mutant adult females showed low sexual receptivity and lordosis behavior, but they can discriminate between sexes (Oboti et al., 2014).

Mice with a targeted disruption of G_ai2_ have a reduced number of vomeronasal neurons that express G_ai2_. Postpartum females and males reduce their aggression toward an intruder, have longer latencies to attack, display few aggressive bouts, and spend shorter periods engaging in aggressive behaviors. Conditional G_ai2_ ablation reduces male aggression toward pups (Trouillet et al., 2019). Sexual inexperienced females display sexual receptivity levels like those found in wild type mice but exhibit a reduced pup retrieval behavior. When G_ai2_ conditional knockout females had sexual experience, they no longer had alterations in pup retrieval or maternal aggression. However, sexual experience did not induce high levels of sexual receptivity as observed in control mice. These knockout females have low neuronal activity in the anterior AOB and the rostral periventricular area of the third ventricle (Trouillet et al., 2021). Male mutant G_ai2_ mice prefer and mate with the female over a gonadectomized male in a mating choice test (Norlin et al., 2003).

The above-described studies using transgenic models further support the contention that the MOB and AOB have complementary roles in reproductive behaviors, contributing to the high plastic capacity of the olfactory system. This brief review of the literature from different species clearly demonstrates the complex interaction between the two early described olfactory systems. We should consider that we are probably dealing with one complex olfactory system with a high degree of plasticity. This plasticity is also evident in the integration of new neurons in adulthood to the OB induced by sexual behavior, which will be described in the next section.

### Neurogenesis in the subventricular zone-rostral migratory stream and olfactory bulbs in response to sexual behavior

As already described, olfaction is crucial for the appropriate selection of a potential mate, for the expression of sexual behavior, and for the species’ survival. Not only does olfactory stimulation induce the formation of new neurons (Peretto and Paredes, 2014), but sexual behavior also induces permanent plastic changes in different parts of the olfactory system inducing the incorporation of new neurons in the adult brain in different species. Depending on the type of stimulation, the incorporation of new cells and neurons is observed in the rostral migratory stream, the AOB, or the MOB. In the following section we will review how this motivated behavior induces long term plastic changes in neurogenesis in different areas of the olfactory system, further confirming the functional interaction and high plastic capability of the olfactory system. Adult neurogenesis has been documented in several species, including rats, mice, hamsters, voles, mole rats, ungulates, birds, fish, amphibians, reptiles, non-human primates, and in humans is still in debate (Portillo et al., 2019).

### Neurogenesis in the olfactory epithelium and vomeronasal epithelium

The olfactory epithelium can continuously renew its neuronal population. The proliferating neuronal precursors and transit-amplifying cells give rise to immature olfactory receptor neurons that differentiate into mature olfactory sensory neurons (Calof et al., 1996, 1998). Pregnant mice showed more new cells in the olfactory epithelium than nonpregnant females. Prolactin can mediate the pregnancy effects over cell proliferation in the olfactory epithelium. Females with intranasal prolactin treatment showed an increase in the number of BrdU-positive cells in the olfactory epithelium, and no significant differences were found between nonpregnant females treated with prolactin and 7 days pregnant mice (Orita et al., 2009).

Vomeronasal sensory neurons are renewed by neurons generated from stem cells at the sensory epithelium. New vomeronasal sensory neurons send their axons to the AOB (De La Rosa-Prieto et al., 2009). The vomeronasal sensory epithelium is a site of ongoing neurogenesis in adult mammals. Positive cells for markers of mature neurons (G_αo_ and G _αi2_) were found after BrdU administration (Martinez-Marcos et al., 2000).

After mating, females form an olfactory memory of their mating partner. Females who do not create this memory cannot recognize their sexual mate, and his pheromones block pregnancy (Bruce effect). The AOS is involved in this olfactory memory (Bellringer et al., 1980; Lloyd-Thomas and Keverne, 1982). Kaba and coworkers demonstrated that females who mated and were treated with ^3^H thymidine on days 1–5 of pregnancy and whose vomeronasal organ was obtained 10 days later had more ^3^H thymidine clusters than females who did not mate. Thus, neurogenesis of the vomeronasal receptors is enhanced during pregnancy, and the authors propose that these new cells can be involved in sexual partner olfactory memory (Kaba et al., 1988). Late pregnancy increases proliferation in the vomeronasal organ epithelium. New cells mature as sensory neurons that project their axons to the olfactory forebrain and can detect peptides and urinary proteins with pheromone activity. Administration of estrogen, but not progesterone, increases vomeronasal progenitor cell proliferation in the vomeronasal epithelium (Oboti et al., 2015).

### Neurogenesis in the subventricular zone-rostral migratory stream and olfactory bulbs

**Figure 1:**
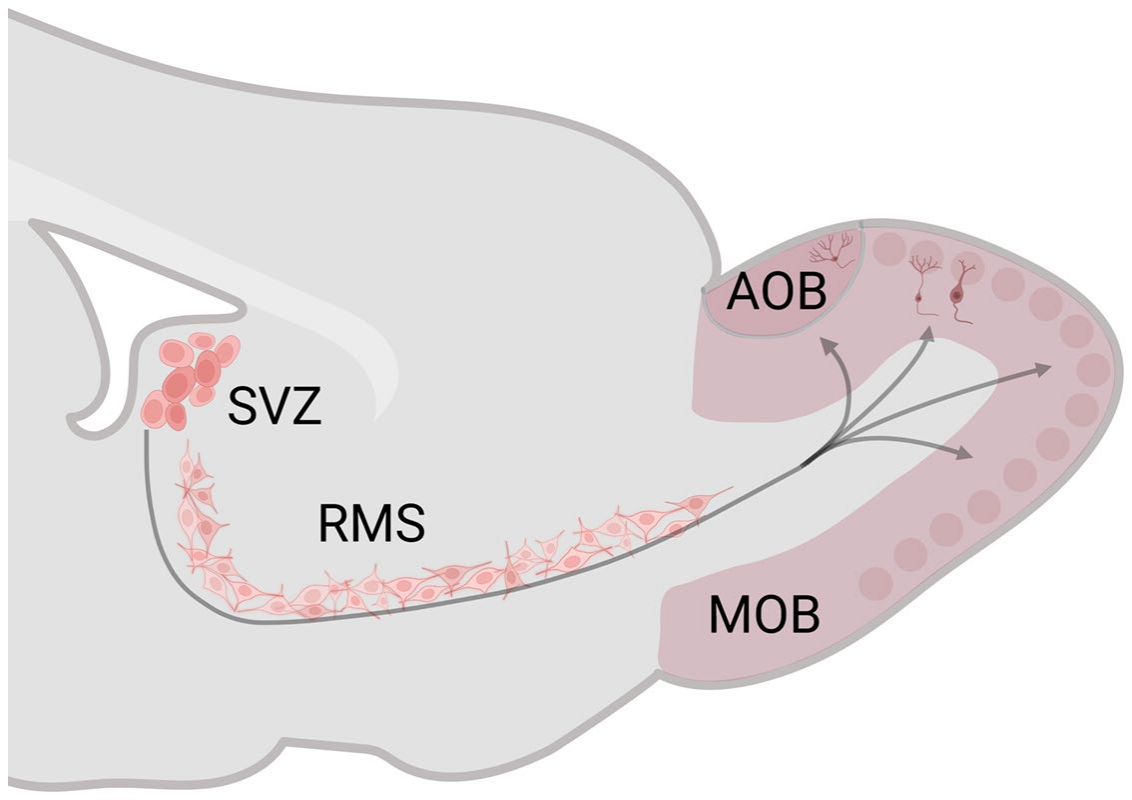
Schematic representation of the subventricular zone (SVZ)-rostral migratory stream (RMS)–olfactory bulb (OB) system. The SVZ generates neural stem/precursor cells, which, during adult life, produce new cells that migrate along the RMS until reaching the OB. Created with BioRender.com.

The subgranular zone of the dentate gyrus (DG) of the hippocampus and the subventricular zone (SVZ) of the lateral ventricle preserve neural stem/precursor cell niches that, during adult life, generate new cells and then migrate to the granular cell layer of the DG and the glomerular and granular cell layer of the MOB and AOB, reviews in Bedos et al. (2018) and Bartkowska et al. (2022). Adult neurogenesis has three principal stages: proliferation, migration, and survival. In the SVZ, after the new cells are born, they migrate tangentially (0–1 week) and radial (1–2 weeks) along the rostral migratory stream (RMS) until reaching the OB (Figure 1). Between 15 and 45 days after birth, the number of new cells decreases to half; in this critical period, sensory stimulation is fundamental for their survival. The new cells that survive integrate into the preexisting OB circuits (45 days after birth) and become functional (Petreanu and Alvarez-Buylla, 2002; Winner et al., 2002; Ming and Song, 2005).

Dentate gyrus proliferation takes around 25 h, fate specification and migration occur 4 days after birth, and cell survival and synaptic integration between 2 and 4 weeks (Ming and Song, 2005). The OB connects with the hippocampus through the entorhinal cortex and perforant path. The entorhinal cortex projects to the DG, a preprocessor of the incoming information to CA3 (Vanderwolf, 1992; Jonas and Lisman, 2014). The OB and hippocampus regulate social and sexual behaviors such as conspecific odor signaling and partner recognition (Catani et al., 2013).

In a series of studies, we evaluated the effect of sexual behavior on the formation of new cells and neurons along the SVZ-RMS-OB system and the hippocampus. Sexual behavior is a motivated behavior necessary for the survival of the species but not for the individual since there are males that do not display sexual behavior despite the fact they are tested repeatedly with receptive females (Portillo et al., 2006, 2010). We have evaluated how sexual behavior induces the formation of new cells and neurons in rats, mice, and prairie voles (*Microtus Ochrogaster*). These new cells and neurons, induced by sexual behavior reaching the main and accessory olfactory bulbs, constitute a clear example of the functional interaction of the AOS and the MOS.

### Neurogenesis in rats

**Figure 2:**
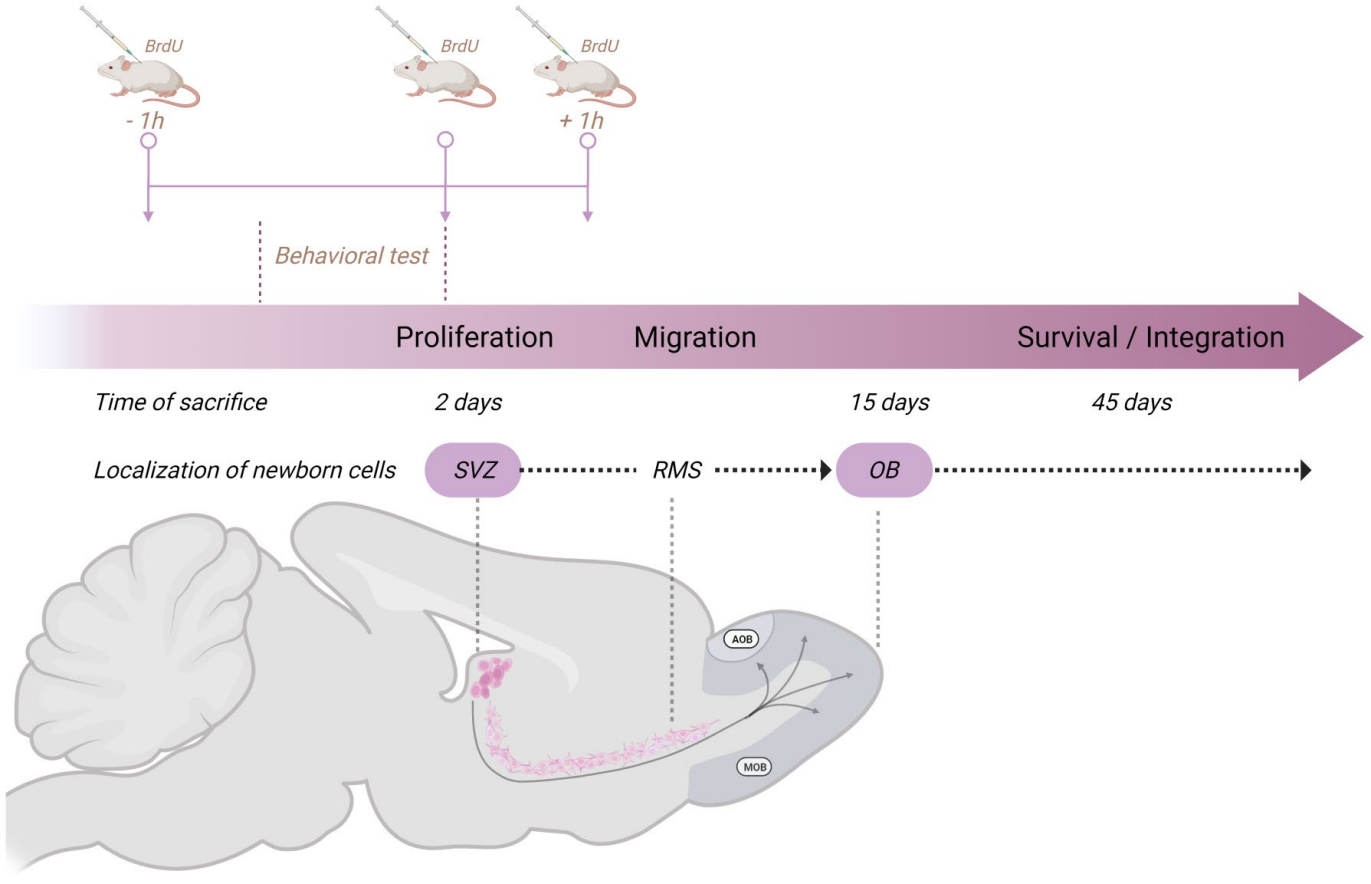
Schematic representation of the general method used in our studies on neurogenesis in the subventricular zone (SVZ)-rostral migratory stream (RMS)–olfactory bulb (OB) system to study the formation of new cells and neurons. The behavioral test lasted 1 h, and we injected the subjects with 5-Bromo-2-deoxyuridine (BrdU) 1 h before, at the end of, and 1 h after the behavioral test. Each dose consists of 100 mg/kg for a total amount of 300 mg/kg. Subjects are then sacrificed 2 days (proliferation), 15 days (migration), or 45 days (survival/integration) after the behavioral test. Created with BioRender.com.

We have evaluated the effects of sexual behavior under paced and non-paced mating conditions. In paced mating conditions, the mating cage is divided by a removable partition with a hole in the bottom that only allows the female to move back and forth from the male compartment, controlling the rate of the sexual stimulation she receives. Paced mating has important physiological and behavioral consequences that favor reproduction. This pattern in which the female controls the rate of sexual stimulation is what occurs in seminatural and natural settings, reviewed in Mier-Quesada et al. (2023) and Paredes and Vazquez (1999). Moreover, when females paced the sexual interaction, a clear positive affective reward state is induced, mediated by opioids (Paredes, 2014). In all our experiments with females, they are ovariectomized and treated with hormones to induce high levels of sexual receptivity (Corona et al., 2011; Arzate et al., 2013). To study the formation of new cells and neurons, we inject 5-Bromo-2-deoxyuridine (BrdU) at three different time points in a dose of 100 mg/kg in such a way that the subjects receive a total of 300 mg/kg. Subjects are injected 1 h before, at the end of, and 1 h after the behavioral test (Figure 2). Following this procedure, we sacrifice the animals at different times after the test to evaluate proliferation (2 days), migration (15 days), and integration (45 days; Figure 2). We then use immunofluorescence and confocal microscopy to assess the colocalization of BrdU and immature and mature neuronal markers (doublecortin and Neun, respectively) and BrdU and GFAP for glial cells. In the experiments, we include different groups: a control group placed alone in the mating cage, females that mated pacing the sexual interaction, females that mated without pacing the sexual interaction, females exposed to sexually experienced males without physical contact and in a cell proliferation study females exposed to banana scent (amyl acetate).

**Figure 3:**
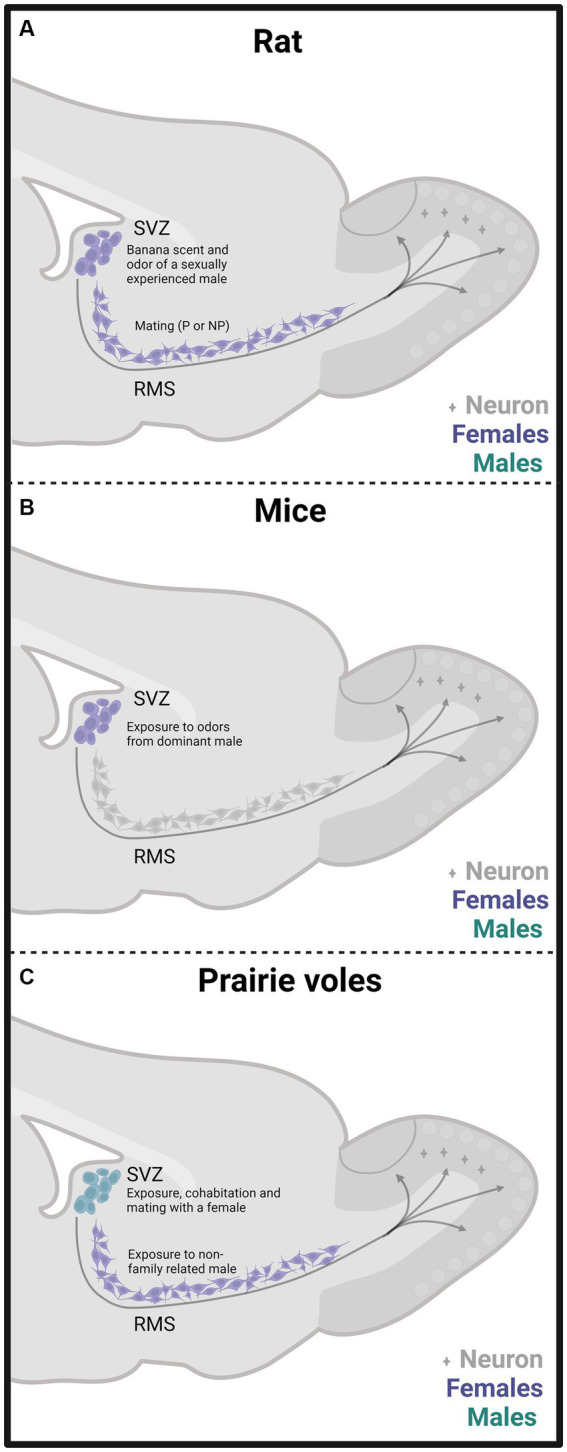
Schematic representation of cell proliferation and neurogenesis in the subventricular zone (SVZ) and rostral migratory stream (RMS) in rats **(A)**, mice **(B)**, and prairie vole **(C)** mated under different conditions. Experiments done in females are depicted in purple and in males in light blue. The stimulation that induces neurogenesis is indicated within the figure. Created with BioRender.com.

**Figure 4:**
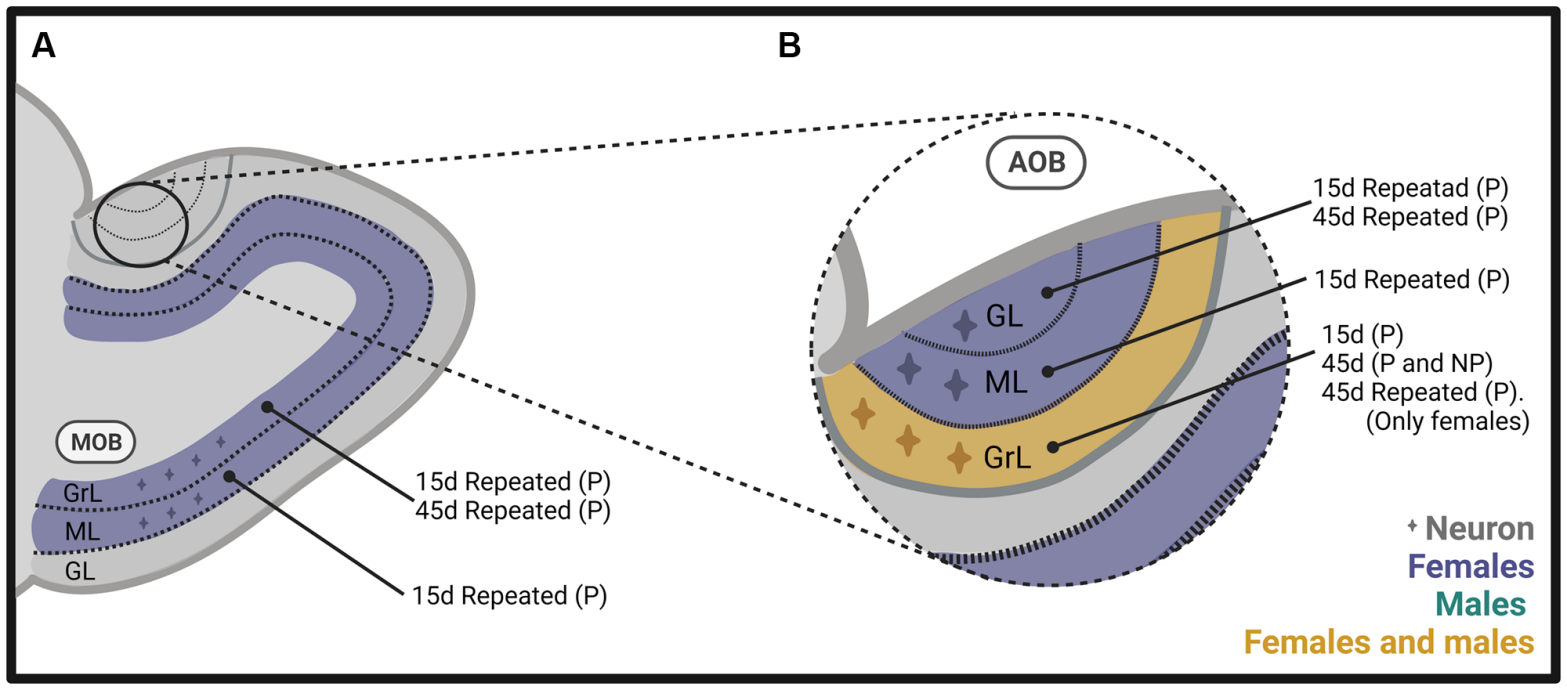
Schematic representation of neurogenesis in **(A)** the main olfactory bulb (MOB) and **(B)** the accessory olfactory bulb (AOB), including the glomerular (GL), mitral (ML), and granular (GrL) layers of male and female rats that mated under different conditions. The stimulation that induces neurogenesis is indicated within the figure. Created with BioRender.com.

In one of our early studies, we sacrificed the animals 2 days, 15 days, or 45 days after mating (Figure 2). We found that the banana scent as the exposure to a sexually experienced male induced a significant increase in the percentage of cells co-labeled for BrdU and doublecortin, a marker of neuroblast and immature neurons in the subventricular zone. We also found more new cells in the groups mated in the anterior RMS (see Figure 3A). These results indicate that olfactory stimulation induces cell proliferation in the SVZ. Mating, paced or non-paced, increases migration in the RMS (Corona et al., 2016). In the groups sacrificed 15 days after mating, we found a significantly higher number of new cells in the granular layer of the AOB in the females that paced the sexual interaction (Figure 4B). In the groups sacrificed 45 days after the sexual behavior test, the groups that mated (paced and non-paced) had a higher percentage of new neurons (double label for BrdU and NeuN) in the anterior granular layer of the AOB (Figure 4B). Some of these cells also expressed Fos after a second mating test 45 days after the first test, suggesting that the newborn cells induced by sexual behavior are activated by mating (Corona et al., 2016).

In follow up studies, we repeated the stimulation to determine if that could modify the expression on neurogenesis since the survival of new neurons reaching the OB is activity-dependent (Yamaguchi and Mori, 2014; Bedos et al., 2018). The methods were similar to those described above (Figure 2), but females mated a total of 4 times, or 10 times, in one or 2 tests per week. We found a significantly higher number of new cells and percentage of new neurons in the granular and mitral layers of the AOB (Figure 4B) and MOB (Figure 4A) in the group of females that paced the sexual interaction. These observations demonstrate that when paced mating is repeated, more new neurons reach the granular layer of the AOB and the MOB (Arzate et al., 2013). When we sacrificed the subjects 45 days after repeated sexual stimulation, we found a significantly higher percentage of new neurons in the granular and glomerular layers of the AOB and the granular layer of the MOB in females that paced the sexual interaction (Figures 4A,B; Portillo et al., 2020). In another study, we extended the period of sexual stimulation by mating the females once weekly for 10 weeks and sacrificed them 45 days after the first test and BrdU administration. We found more cells in the glomerular layer of the AOB and more neurons in the granular layer of the MOB in the group of females that paced the sexual interaction (Figures 4A,B; Alvarado-Martinez and Paredes, 2018). Together, these results indicate that depending on the frequency of sexual stimulation, more cells and neurons reach the main and accessory olfactory bulbs. As testing is repeated, more neurons reach the OB.

We also evaluated the effects of mating on neurogenesis in the DG of females that mated pacing or not the sexual interaction 15 days after the BrdU administration. We found more cells in the ventral DG in the groups that mated in pacing or non-pacing conditions compared to the control group. In the females that paced the sexual interaction for four sessions, more cells were found in the dorsal and ventral DG compared to all other groups (Portillo et al., 2019).

The neurogenesis induced by sexual behavior appears to be opioid dependent. The administration of the opioid antagonist naloxone did not modify neurogenesis in a group that did not mate. However, it blocked the increased number of new cells and neurons in females that paced the sexual interaction (Santoyo-Zedillo et al., 2017). The role of opioids is consistent with the observations that sexual behavior induces a positive affective state in males and females mediated by opioids (Paredes, 2014).

To summarize, our results in female rats indicate that exposure to a sexually experienced male or banana scent induces the proliferation of new cells in the SVZ. Both mating types increase the migration of new cells in the RMS (Figure 3A). One session of paced mating induces new cells and neurons in the granular layer of the AOB. In contrast, repeated paced mating induces more cells in the mitral layer of the AOB and cells and neurons in the granular and mitral layers of the MOB (see Figures 4A,B). It appears, then, that initially, there is a plastic change in the granular layer of the AOB, resulting in adult neurogenesis. If the sexual stimulation is repeated, other layers of the AOB and the MOB integrate new cells and neurons. If the sexual stimulation is repeated after the first mating test, the final fate of the new cells and neurons is not modified.

We found similar results in male rats as those observed in females. The method was like that used in females (Figure 2). When males were sacrificed 15 days after mating and BrdU injection, we found a higher number of cells in the granular layer of the AOB when they ejaculated one or three times, pacing the sexual interaction (Figure 4B). No changes in the number of cells and neurons were observed in males that could not control the sexual stimulation (the females paced) and in males exposed to sexually receptive females (Portillo et al., 2012). When males were sacrificed 45 days after ejaculating three times, pacing the sexual interaction, a higher number of cells and neurons was observed in the granular layer of the AOB (Figure 4B), but no changes were observed in the MOB (Unda et al., 2016). Some studies have evaluated neurogenesis in the hippocampus induced by sexual behavior in males, but their description is beyond the scope of the present manuscript. The interested reader can find information in Bedos et al. (2018).

### Prairie voles

The relevance of adult neurogenesis induced by sexual behaviors in socially monogamous rodents has also been evaluated. *Microtus ochrogaster* (prairie vole) is a cricetid with a socially monogamous mating strategy. Adult females are not sexually receptive until active sniffing of odors from non-family-related males induces estradiol release and sexual receptivity (Cohen-Parsons and Carter, 1987). In prairie voles, cohabitation with mating for 6 h or without mating for 24 h induces an enduring pair bond (Williams et al., 1992; Wang et al., 1997). This socio-sexual behavior is characterized by a preference for the mating partner, mate guarding, cohabitation in the nest, sharing and defending their territory, and biparental care of their offspring; reviews in Gobrogge (2014) and Walum and Young (2018).

Since a pair bond implies the memory of the sexual partner, adult neurogenesis can be involved in this process. Dr. Wang’s research group was the first to evaluate in female voles the effects of sexual behavior that led to pair bonding over adult neurogenesis. Their study demonstrated that in sexually naïve females, estrous induction by exposure to non-family related males increases cell proliferation in the RMS by 90% and, interestingly, 80% of the new cells express markers of neuronal lineage commitment. Cell proliferation depends on estradiol since ovariectomized females without hormone replacement exposed to a male did not increase cell proliferation (Smith et al., 2001).

In prairie voles, the amygdala and hypothalamus integrate new cells in response to sexual behaviors. In female voles, cohabitation with mating increases cell proliferation in the amygdala and hypothalamus compared to females in social isolation, but no changes were found in the SVZ. In addition, pregnant females that cohabitated with their sexual partner 3 days after parturition increased the number of new cells that survive in the amygdala, but no changes were observed in the OB and DG of the hippocampus (Fowler et al., 2002).

Our research group demonstrated that male prairie voles that cohabitated and mated with a female and those exposed to another male or a female showed an increase in the percentage of neuron precursor cells in the SVZ (Figure 3C). Also, these males showed fewer precursor cells in the RMS’s medial region than control males. We also found that social cohabitation with mating and social exposure to a female increases cell proliferation in the DG of the hippocampus (Castro et al., 2020).

**Figure 5:**
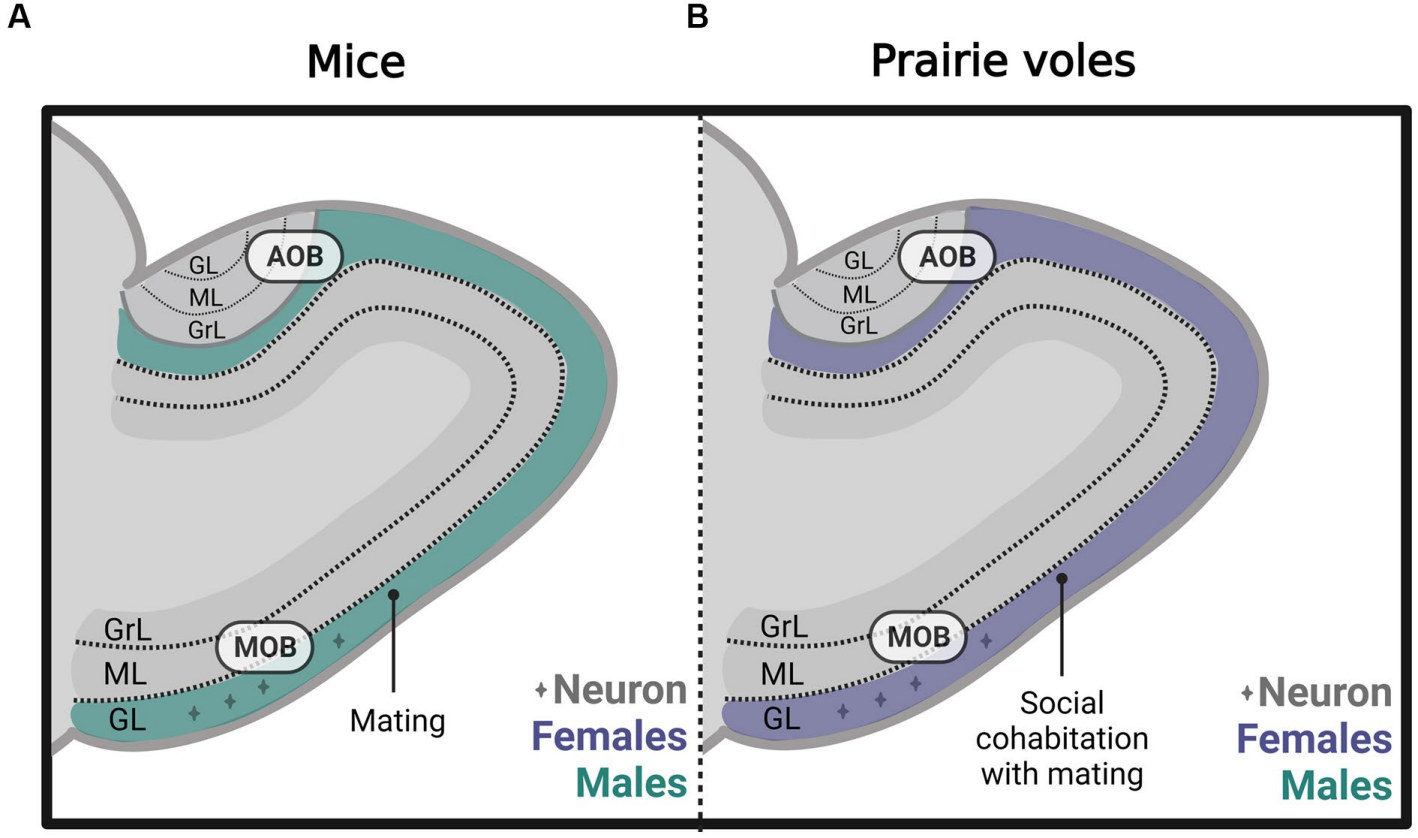
Schematic representation of neurogenesis in the main (MOB) and accessory olfactory bulb (AOB) in **(A)** mice and **(B)** prairie voles, including the glomerular (GL), mitral (ML), and granular (GrL) layers that mated under different conditions. Created with BioRender.com.

Subsequently, in male and female prairie voles, we evaluated the effects of socio-sexual stimuli on cell survival. We found that social cohabitation with mating in females increases the number of new neurons that survive in the glomerular layer of the MOB (Figure 5B) and cell survival in the DG of the hippocampus. In male voles, social cohabitation with mating and social exposure decreases cell proliferation in the glomerular layer of the MOB (Castro et al., 2022). Further studies are needed to determine the physiological relevance of the new cells in mate recognition, pair bond maintenance, and parental care.

In Mandarin voles (*Microtus mandarinus*), another social-monogamous species, adult neurogenesis plays a relevant role in social recognition. Mandarin voles pups at postnatal days 14–21 display high levels of social attachment to their parents. Paternal separation at this age impairs social recognition in male and female adult Mandarin voles. Interestingly pre-weaning paternal separation decreases the number of new cells and mature neurons in females and the number of new immature neurons in males in the DG of the hippocampus. No effects were found in the new glial cells (He et al., 2018).

### Mice

Female mice exposed to odors from dominant males showed increased cell proliferation in the SVZ (Figure 3B) and DG of the hippocampus and enhanced number of immature and mature neurons in the OB and DG. MOE lesions with ZnSO4 inhibited SVZ and DG cell proliferation induced by male-dominant odors, highlighting the relevance of the MOS (Mak et al., 2007). However, the significance of the AOS was not evaluated. Furthermore, the administration of the antimitotic drug cytosine arabinoside in female mice inhibits their preference for dominant male odors (Mak et al., 2007). Adult neurogenesis induced by male pheromones is probably mediated by the male major urinary protein darcin (MUP20). Darcin increases cell proliferation in the SVZ and the percentage of immature neurons in the DG of the hippocampus (Hoffman et al., 2015).

Nunez-Parra and coworkers demonstrated that in sexually naïve female mice, male odors increase the number of new cells in the AOB. This effect was specific since female scents did not modify neurogenesis (Nunez-Parra et al., 2011). In male mice, our research groups demonstrated that sexual behavior increases the percentage of new cells that differentiate into neurons in the glomerular layer of the MOB (Figure 5A). However, no differences were found in the AOB or the granular cell layer of the MOB (Velazco-Mendoza et al., 2019).

## Conclusion

The interconnection between the AOS and the MOS is evident at different levels, including behavioral, anatomical, and physiological. The induction of new cells and new neurons in the olfactory bulbs induced by sexual behavior in males and females is another example of this interaction. In the female rat, odors of sexual origin or another type of odors induce cell proliferation in the SVZ, while mating (paced or non-paced) increases the number of new cells in the RMS. One paced mating session induces more cells 15 days after mating. When the stimulation is repeated, more cells reach different layers of the AOS and MOB. These plastic changes could be mediated by three factors: The frequency of the stimulation (number of mating sessions), the temporal presentation of the stimulus (number of sessions in 15 days or 45 days), and the release of opioids induced by sexual behavior. We suggest that changes induced by mating support the hypothesis that the AOS and the MOS are part of a large olfactory system with a high plastic capability, allowing species to adapt to the environment.

## Author contributions

ZM and WP: writing, review, and editing. RP: conceptualization, writing, review, and editing. All authors contributed to the article and approved the submitted version.
